# Newly Identified Roles of PML in Stem Cell Biology

**DOI:** 10.3389/fonc.2013.00050

**Published:** 2013-03-14

**Authors:** Kyoko Ito, Keisuke Ito

**Affiliations:** ^1^Ruth L. and David S. Gottesman Institute for Stem Cell and Regenerative Medicine Research, Albert Einstein College of MedicineBronx, NY, USA; ^2^Department of Cell Biology, Albert Einstein College of MedicineBronx, NY, USA; ^3^Department of Medicine, Albert Einstein College of MedicineBronx, NY, USA; ^4^Albert Einstein Cancer Center, Albert Einstein College of MedicineBronx, NY, USA

**Keywords:** PML, stem cells, metabolism, stem cells and differentiation, breast cancer

## Abstract

It has long been believed that the tumor suppressor promyelocytic leukemia (PML), the core component of the nuclear substructures known as the PML-nuclear bodies, plays a key part in acute PML (APL), as it is first cloned at the breakpoint of the t(15;17) translocation typical of that disease. Research over the past decade, however, has radically changed our view of how this tumor suppressor is regulated, how it can be therapeutically targeted, and how it functions in a number of tissue systems. One noteworthy recent study, for instance, revealed that PML regulates the activation of fatty acid metabolism, and that this metabolic reprograming plays an essential role in cancer biology and stem cell biology through the control it exerts over stem cell fate decisions. These findings sparked exciting new investigations of PML as a critical “rheostat” responsible for fine-tuning tissue homeostasis, and thus created at the intersection of cancer and stem cell biology a new field of study with important therapeutic implications.

## Multi-Functional Protein, PML

**Figure 1:**
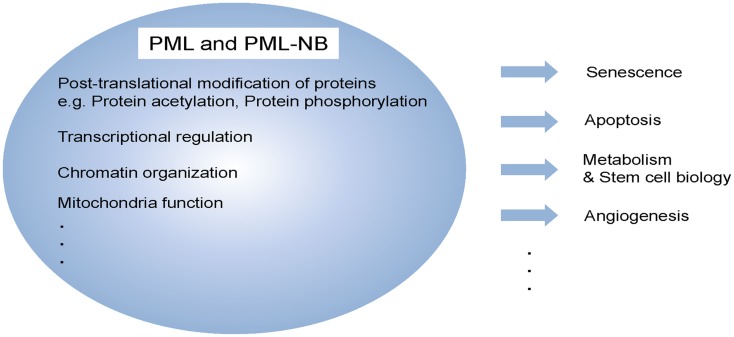
**Multi-functional protein, PML**. PML and/or PML-NBs regulate a diverse of cellular functions, including senescence, apoptosis, metabolism, stem cell function, and angiogenesis. These functions are achieved by different biochemical means such as post-translational modification, transcriptional regulation as well as mitochondria function.

The tumor suppressor promyelocytic leukemia (PML) was first identified as a fusion partner of human retinoic acid receptor alpha (RARα) as the result of a chromosomal translocation discovered in acute PML (APL) (de The et al., 1991; Kakizuka et al., 1991). It was already known to be the essential component of the multi-protein sub-nuclear structures commonly referred to as the PML-nuclear bodies (NBs) (Zhong et al., 1999), which control a variety of post-translational modifications (sumoylation, phosphorylation, or acetylation) of a range of recruited partner proteins (Figure 1) (Lallemand-Breitenbach and de The, 2010). Unlike the well-established roles of PML and PML-NBs in solid tumors and leukemia pathogenesis [which are effected through modulation of the activity of p53 (Bernardi et al., 2004), Atk (Trotman et al., 2006), mTOR, HIF1α (Bernardi et al., 2006), or mitochondrial regulatory pathways (Giorgi et al., 2010)], little is known about the role of this tumor suppressor in stem cell biology or the mechanisms of its regulation of cancer metabolism.

## PML in Stem Cells

The phosphoinositide 3-kinase (PI3-kinase) signaling pathway controls cell proliferation, growth, and survival by integrating numerous upstream signals, including growth factors, nutrients, and oxygen status. Critical modulators and effectors of this pathway, such as PTEN (phosphatase with tensin homology, which is deleted on chromosome 10) and the forkhead O (FoxO) family, have recently been implicated in the stem cell biology of the hematopoietic system (Trotman et al., 2006; Yilmaz et al., 2006; Zhang et al., 2006; Miyamoto et al., 2007; Tothova et al., 2007; Guo et al., 2008; Ito et al., 2008), where improper Akt-mTorc1 signaling can lead to profound defects in the maintenance of hematopoietic stem cells (HSCs). Indeed, growing evidence suggests that tight regulation of this signaling pathway is essential to the maintenance of normal hematopoiesis.

Promyelocytic leukemia had previously been identified as a negative regulator of Akt and mTOR pathway at multiple levels. We recently defined a critical role for PML in the stem cells of the hematopoietic system (Ito et al., 2008), and demonstrated that deletion of *Pml* leads to loss of quiescence in HSCs, resulting in their transient amplification and subsequent exhaustion.

We further defined a critical role for PML in the maintenance of leukemia-initiating cells (LICs) in disease models of chronic myeloid leukemia (CML), and presented a new therapeutic approach based on targeting quiescent LICs by pharmacological inhibition of PML. PML is often elevated in patients with CML, and these higher levels have been shown to correlate with poor clinical outcomes. In addition, *Pml*-deficient LICs become exhausted with time, and are incapable of generating CML in transplanted animals. Interestingly, inhibition of PML by arsenic trioxide (As_2_O_3_) disrupted LIC maintenance and sensitized LICs to anti-leukemic therapy with little adverse effect on normal HSCs (Ito et al., 2008).

All these findings strongly support an essential role for PML in LIC biology, and suggest there could be a window for effective CML therapy via PML-targeting. This in turn inspired us to propose that PML-lowering drugs should be used temporarily at leukemia onset, along with, or followed by, standard-of-care regimens. However, questions remain regarding the molecular mechanism underlying the effect of PML on HSC maintenance.

## A New Role for PML in Metabolic Function

Normal cells rely primarily on mitochondrial oxidative phosphorylation to produce adenosine triphosphate (ATP), which maintains cellular viability and functions by utilizing three major bioenergetic fuels: glucose, glutamine, and fatty acids.

Many cancer cells, however, have been shown to rely on aerobic glycolysis for survival, growth, and the efficient production of biomass [recent studies indicate that some cancer cells depend on glutamine as well (Dang, 2010)]. This altered metabolism in cancers is the result of either oncogene activation or the loss of tumor suppressor genes in multiple signaling pathways, including the PI3-kinase and Myc pathways (Deberardinis et al., 2008; Vander Heiden et al., 2009; Dang, 2010). Relatively little is known, however, about the role of fatty acids as a bioenergetic fuel in the growth and survival of cancer cells.

It is established, however, that in breast cancer cells, fatty acid oxidation (FAO) functions as the source of ATP once epithelial glandular structures lose their normal architecture and cancer cells start to proliferate aberrantly, migrating far from the extracellular matrix and undergoing metabolic stress (Schafer et al., 2009). This loss of attachment results in the inhibition of glucose uptake and glycolytic influx, while the ensuing decrease in NADPH leads to increased reactive oxygen species (ROS), which inhibits fatty acid catabolism. Enhancing FAO by antioxidant treatment increases the survival capacity of these cells upon loss of attachment.

As part of this developing picture of how breast cancer is fueled, we have recently identified a tumoral metabolic reprograming for PML that is central to breast cancer cell survival. As we have noted, FAO is known to play an important role in the metabolic challenge unleashed by loss of attachment in breast cancer cells (Carracedo et al., 2012). We have shown that under these conditions, PML acts as both a negative regulator of peroxisome proliferator-activated receptor (PPAR) γ co-activator 1α (PGC1α) acetylation and an activator of PPAR-FAO signaling. The PML-PPARδ-FAO pathway in primary breast cancer cells grown in methylcellulose renders these cells resistant to anoikis, which results in luminal filling in a 3D basement membrane breast culture model (Carracedo et al., 2012). Mechanistically, PML-NBs recruit SIRT1 deacetylase to greatly decrease PGC1A acetylation, enhancing its co-activator function on PPARδ signaling and triggering the expression of enzymes which control FAO. PML is consistently enriched in triple-negative cases, and *PML* expression in breast cancer correlates with both activated PPAR signaling and reduced time to recurrence, a gene signature of poor prognosis.

Taken together, these findings have clear therapeutic implications, suggesting that targeting PML may represent a novel therapeutic avenue in the treatment of triple-negative breast cancer. At the same time, they raise the issue of whether a metabolic function of PML can be similarly described and targeted as a common cellular mechanism in other tissue systems (e.g., hematopoiesis) as well as in stem cell biology.

## Fatty Acid Metabolism and Hematopoietic Stem Cell Homeostasis

Under normal conditions, HSCs remain in a quiescent state and respond to environmental insults by entering the cell cycle, dividing, and giving rise to multi-potent progenitors (Lemischka and Moore, 2003; Arai et al., 2004; Fuchs et al., 2004; Wilson and Trumpp, 2006; Morrison and Spradling, 2008). Over the past few years, the metabolism of these essentially quiescent cells has been the focus of many studies (Gan et al., 2010; Gurumurthy et al., 2010; Nakada et al., 2010; Simsek et al., 2010; Takubo et al., 2010; Suda, 2011). The most recent studies have focused largely on glycolysis and energy homeostasis, however, while the contribution of fatty acid metabolism to HSC maintenance has remained relatively unexplored (Suda, 2011).

We modeled our investigation of these questions by first translating the known role that PML plays in breast cancer to a similar possible role in stem cell biology. We then demonstrated that PML activates PPARδ, a nuclear receptor that plays a key role in stem cell maintenance. The fact that PPARδ levels and signaling output are reduced during the differentiation of HSCs inspired us to further elucidate the role of fatty acid metabolisms in HSC maintenance.

*In vivo* and *in vitro* assays soon demonstrated that conditional loss of *Ppard* profoundly affects quiescence, maintenance, and function of HSCs, while treatment with PPARδ activators increases HSC maintenance and function. Conversely, defects in *Pml^−/−^* HSCs were partly rescued by PPARδ agonists. PPARs are central regulators of metabolism and control mitochondrial function, in particular FAO (Braissant et al., 1996; Michalik et al., 2006; Takahashi et al., 2007). Pharmacological abrogation of FAO mimics the consequence genetic loss of *Pml*, resulting in the ultimate loss of HSC maintenance, and antagonizing the beneficial effects achieved by PPAR agonists in terms of improving HSC function (Ito et al., 2012).

Several essential pathways in HSC maintenance, including FOXO-oxidative stress (Miyamoto et al., 2007; Yalcin et al., 2008), hypoxia-glycolysis (Simsek et al., 2010; Takubo et al., 2010), the LKB1-mTOR pathway (Gan et al., 2010; Gurumurthy et al., 2010; Nakada et al., 2010), are clearly implicated in energy metabolism. We now have direct evidence identifying fatty acid metabolism as a critical factor in the self-renewal of HSCs.

## PML-PPARδ Pathway and FAO Control HSC Asymmetric Division

Stem cells have two specific functions; self-renewal and pluripotency. One of the central tasks of stem cell biology is to understand the modes and mechanisms that regulate self-renewal and commitment of stem cells, as alterations in this equilibrium impact hematopoietic homeostasis and maintenance. Identifying the factors regulating this process is of high biological and therapeutic relevance.

Possible division patterns of stem cells include asymmetric division, where an HSC gives rise to either two distinct daughter cells, one HSC and one committed progenitor cell (an outcome which results in HSC maintenance), or symmetric division, which can result in either two HSCs (HSC expansion) or two committed progenitors (HSC exhaustion). Asymmetric cell division has recently been suggested as a regulator of cell-fate decisions in the mammalian hematopoietic system, with crucial roles in stem cell renewal to ensure that a fraction of the daughter cells retains stem cell features while replenishing the compartment (Suda et al., 1983, 1984; Lansdorp, 1997; Metcalf, 1998; Morrison and Kimble, 2006).

**Figure 2:**
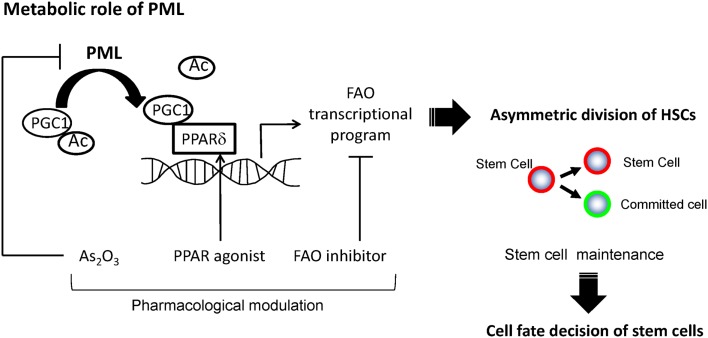
**A model for regulation of asymmetric division by PML-PPARδ-fatty acid oxidation**. PML increases the fraction of de-acetylated PGC1α, and leads to the activation of PPAR signaling and fatty acid oxidation (FAO). These metabolic changes promote HSCs toward the fate of asymmetric cell division, leading to their maintenance.

We studied the symmetry of cell divisions of HSCs using a binary immunophenotypical assay that takes advantage of two surface markers, Tie2 and CD48 (Arai et al., 2004; Kiel et al., 2005; Ito et al., 2012). We found that in single cell culture assays, more than 40% of purified HSC compartments asymmetrically divided, giving rise after the first cell division to two distinct daughter cells, Tie2^pos^CD48^neg^ (HSC) and Tie2^neg^CD48^pos^ (a committed cell). This resulted in the discovery of a link between the regulation of fatty acid metabolism by PML and control of HSC division (Ito et al., 2012). Depletion of *Ppard* or *Pml*, as well as mitochondrial FAO inhibition, results in a symmetric commitment of HSC daughter cells *in vitro* and *in vivo*. On the other hand, pharmacological PPARδ activation increases asymmetric division, thereby supporting the maintenance of the HSC population (Figure 2). Importantly, each of the sequential key steps in this cascade of fatty acid metabolism can be pharmacologically intervened: PML by interferons and As_2_O_3_, PPARδ by existing agonists available for human use, and FAO by specific inhibitors of the mitochondrial enzymes responsible for fatty acid catabolism.

These findings strongly suggest that a metabolic requirement exists for the exquisite regulation of asymmetric division of HSCs, which sets the baseline for metabolic reprograming during the process of stem cell differentiation. These evidences not only act as bridge between nuclear organization, transcriptional control, and lipid metabolism in decisions underlying asymmetric cell division, but also have major implications for therapeutic manipulation of HSCs.

## Clinical Implication and Future Directions

Our study has uncovered a role for the PML–PPAR-δ–FAO pathway in the control of HSC asymmetric division and cell maintenance; these findings may open new therapeutic avenues through the manipulation of normal HSCs. For instance, the benefit of PPARδ on HSC function implies the therapeutic potential of PPARδ activators, especially in conditions where stem cell function needs to be maximized, as is the case with hematopoietic recovery after bone marrow transplantation.

However, there are several issues with this approach still to be discussed and elucidated in greater depth in future studies.

First, while the potential therapeutic power of pharmacological inhibitors of FAO may lie in their ability to exhaust stem cells, it remains to be determined whether PML exerts such a role in *leukemia* stem cells (or LICs). We have previously shown that A_2_O_3_, a PML-targeting compound, effectively induces quiescent LICs to enter the cell cycle (Ito et al., 2008). The demonstrated fact that *Pml*-deficient HSCs exhibit reduced PPAR signaling and FAO tempts us to propose that the use of pharmacological inhibitors of FAO to promote LIC exhaustion may represent an alternative therapeutic strategy to targeting differentiated leukemic cells (Samudio et al., 2010). Further extensive investigations will confirm whether FAO inhibitors and/or a combination of these drugs with compounds targeting the proliferating leukemic cell pool will have the power to effectively contain or reverse this disease.

Second, the fact that PML appears to be the upstream regulator of this key regulatory cascade raises the issue of how PML and PML-NBs are themselves regulated in stem cells. Third, these metabolic requirements may parallel similar requirements in other tissue stem cells (e.g., neural stem cells) as well as human hematopoiesis. Lastly and perhaps most importantly, it is still unknown how the metabolic changes induced by FAO can directly control asymmetric HSC division. It will also be of interest to determine how PML-PPAR-FAO pathways asymmetrically segregate cellular organelles as well as cell-fate determinants. Specific “druggable” targets with therapeutic value are likely to be determined by directly linking the mechanisms of cell metabolism and stem cell division.

On the basis of what we have discussed, it is clear that PML is a unique protein that regulates diverse cellular functions, and that we now understand in greater detail the mechanisms it regulates, knowledge which may soon be translated into effective treatment modalities. Open questions remain, however, regarding PML and its functions, that will no doubt motivate further investigation of this intriguing tumor suppressor.

## Conflict of Interest Statement

The authors declare that the research was conducted in the absence of any commercial or financial relationships that could be construed as a potential conflict of interest.
